# Kefir and Its By-Products Supplementation Reduces Inflammation and Oxidative Stress, Improves Intestinal Barrier Integrity, and Modulates the Gut Microbiota in Animal Models of Inflammatory Bowel Disease: A Systematic Review

**DOI:** 10.1007/s12602-026-10948-5

**Published:** 2026-03-03

**Authors:** Maria Alice Spadarotto Neves, Rayanne Santos de Paulo, Josefina Bressan, Solange Silveira Pereira, Ana Claudia Pelissari Kravchychyn, Helen Hermana Miranda Hermsdorff

**Affiliations:** 1 Laboratory of Clinical Nutrition, Department of Nutrition and Health, Universidade Federal de Viçosa (UFV), Viçosa, MG Brazil; 2 Laboratory of Clinical Analysis and Genomics, Department of Nutrition and Health, Universidade Federal de Viçosa (UFV), Viçosa, MG Brazil; 3 Laboratory of Energy Metabolism and Body Composition, Department of Nutrition and Health, Universidade Federal de Viçosa (UFV), Viçosa, MG Brazil

**Keywords:** Kefir, Inflammatory bowel diseases, Animal model, Ulcerative colitis, Crohn’s disease, Gut health

## Abstract

Kefir is a beverage obtained by fermenting milk or sugary solutions with a symbiotic community of bacteria and yeasts, presenting promising antimicrobial, antioxidant, and immunomodulatory properties. This systematic review aimed to synthesize evidence from preclinical studies evaluating the effects of kefir or its by-products on biomarkers of inflammation, oxidative stress, and gut health in animal models of IBD. A systematic review was conducted in accordance with PRISMA guidelines, utilizing the PubMed/MEDLINE, Web of Science, Embase, and Scopus databases. The quality of the studies was assessed using SYRCLE’s Risk of Bias tool. Sixteen experimental studies were included, comprising 585 rodents with chemically induced colitis. The interventions included traditional milk kefir, rice and water kefir, as well as isolated microorganisms and kefir-derived supernatants. Most studies reported reductions in inflammatory cytokines (TNF-α, IL-1β, IL-6) and inflammatory enzymes (iNOS, COX-2, MPO), along with increases in anti-inflammatory cytokines (IL-10, IL-4). Reductions in MDA and H₂O₂ were reported, supporting the antioxidant effects of kefir and its derivatives. Changes in antioxidant enzyme activity, including SOD, were also observed. In addition, kefir modulated gut microbiota composition, upregulated the expression of tight junction proteins, and influenced immune and molecular signaling pathways. Improvements were also observed in clinical parameters of IBD models, including disease activity index, rectal bleeding, and histological damage. Kefir and its derivatives exhibit beneficial effects on inflammation, oxidative stress, gut permeability, and immune modulation in animal models of IBD, suggesting a potential alternative for treating these diseases in humans. Although the findings are promising, heterogeneity among study protocols and methodological limitations highlight the need for further studies. Registration PROSPERO number: CRD420251062931.

**Supplementary Information:**

The online version contains supplementary material available at https://doi.org/10.1007/s12602-026-10948-5.

## Introduction

Inflammatory bowel diseases (IBD) are chronic inflammatory disorders of the gastrointestinal tract that have increased markedly in recent years, currently affecting an estimated 5 million individuals worldwide. IBD is primarily classified into two major subtypes: Crohn’s disease and ulcerative colitis [1, 2]. Ulcerative colitis is confined to the colon and involves superficial inflammation of the mucosa, which spreads continuously from the rectum upward and may result in ulcerations, severe bleeding, toxic megacolon, and fulminant colitis. In contrast, Crohn’s disease can affect any region of the digestive tract in a discontinuous, patchy manner and is characterized by transmural inflammation, often leading to strictures, fistulas, and other complications [1, 2].

These diseases cause debilitating symptoms and reduce patients’ quality of life, such as diarrhea, abdominal pain, weight loss, and fecal bleeding. Although conventional therapies using aminosalicylates, corticosteroids, immunomodulators, and biological agents are available, their effectiveness after long-term treatment remains limited. As a result, growing interest has emerged in exploring complementary and alternative approaches that modulate intestinal inflammation and support gut health more effectively [2–4].

In this context, fermented products have attracted attention for their potential to improve gastrointestinal health. Kefir, in particular, has been highlighted due to its complex microbiota and fermentation-derived bioactive compounds, which are associated with antimicrobial, antioxidant, and anti-inflammatory properties [3, 5]. Kefir is traditionally produced from kefir grains, which consist of a symbiotic community of lactic acid bacteria, acetic acid bacteria, and yeasts, including *Lactobacillus kefiri*,* Leuconostoc*,* Lactococcus*,* Acetobacter*,* Kluyveromyces marxianus*, and *Saccharomyces spp* [3, 5].

Depending on the fermentation substrate, commonly milk (milk kefir) or sugar water (water kefir), the resulting beverage and its by-products may vary considerably in microbial composition, metabolic profile, and functional properties. These differences may influence its probiotic potential and its effects on gut microbiota, immune modulation, and mucosal integrity [3, 5].

Despite increasing evidence from studies, no comprehensive synthesis currently exists that systematically evaluates the effects of kefir and its derivatives on intestinal health, particularly in the context of IBD. Therefore, this systematic review aims to critically assess the available evidence from preclinical studies investigating the effects of kefir consumption on inflammatory, oxidative stress, histological, and microbiota-related outcomes in IBD.

## Materials and Methods

### Protocol and Registration

The review protocol was registered in the International Prospective Register of Ongoing Systematic Reviews (PROSPERO) (registration number: CRD420251062931), available from https://www.crd.york.ac.uk/PROSPERO/view/CRD420251062931. The review followed the PRISMA method, and the checklist is available in supplementary Table 1.

### Eligibility Criteria

Experimental studies with rodents, such as mice or rats, that evaluated the effects of kefir or its by-products administration on IBD, including ulcerative colitis or Crohn’s disease, were considered eligible for review. To guide the selection of studies and define the main research question, the PICOS framework was applied (P = Population; I = Intervention; C = Comparison; O = Outcomes; S = Study design). Based on this strategy, the central question formulated was: “Does supplementation with kefir or its by-products improve or reduce inflammation or intestinal health in animal models of inflammatory bowel disease?” (Table 1).

**Table 1:** Eligibility criteria of included studies based on PICO framework

Parameter	Inclusion criteria
**P**opulation	Rodents (rats or mice) with experimentally induced IBD (Crohn’s disease or ulcerative colitis)
**I**ntervention	Consumption of kefir and/or its isolated by-products (like *Lactobacillus*, extracellular vesicles derived from *Lactobacillus*, all from kefir)
**C**omparison	Administration of other foods or water
Outcomes	Primary: Inflammatory markers (cytokine levels of TNF-α, IL-1β, IL-4, IL-6, IL-10; expression of inflammatory enzymes cyclooxygenase-2, inducible nitric oxide synthase, and myeloperoxidase), clinical and histological scores (disease Activity Index, presence of diarrhea, rectal bleeding, body weight loss, and histological score). Secondary: oxidative stress biomarkers (malondialdehyde, glutathione, superoxide dismutase, catalase, and glutathione peroxidase), immunological and molecular outcomes (presence of immune cells, activation of signaling pathways, and markers of apoptosis), gut health indicators (gut microbiota composition, expression of tight junction proteins)
**S**tudy design	Experimental studies

Exclusion criteria were studies with just human participants or in vitro experiments, or if they use animal models that don’t exhibit IBD, or any other animals that are not rodents, rats, mice; studies that contain administration of kefir or its by-products only combined with other substances, which don’t evaluate them in isolation; studies without a clear description of the control group intervention.

### Search Strategy

The article selection process was conducted using the databases PubMed/MEDLINE, Web of Science, Embase, and Scopus. These databases were selected because they are robust, comprehensive, and widely recognized for indexing a large volume of high-quality scientific literature, including extensive coverage relevant to the topic of this systematic review. Two reviewers (M.A.S.N. and R.S.P.) carried out the searches independently and in parallel in May 2025, without applying any language or publication date filters. The search terms were derived from Medical Subject Headings (MeSH), Health Sciences Descriptors (DeCS), and Emtree terms, all of which are in English. Boolean operators “OR” and “AND” were applied to combine terms appropriately. Other databases, such as the Cochrane Library, were not included because they primarily focus on clinical trials, whereas this review included only experimental studies. The complete search strategy for all databases is provided in supplementary Table 2. The last search was conducted in May 2025.

### Study Selection and Data Extraction

Studies were selected through a three-step screening of titles, abstracts, and full texts, conducted independently and concurrently by two researchers (M.A.S.N. and R.S.P.), according to the inclusion criteria. The search results were imported into the Rayyan QCRY^®^ software to remove duplicates. The selection process was then carried out using the same software by the same two reviewers working independently, blindly, and in parallel. Any discrepancies following full-text review were resolved by involving a third author (A.C.P.). Once the eligible studies were confirmed, a summary table was compiled (Table 3). Each study was assessed based on its design, kefir intervention, and analysis of health outcomes. The structure of the summary table was defined by mutual agreement among the authors and included the following elements: author and year of publication, study design and duration, intervention characteristics (e.g., kefir type and dose), sample characteristics, study groups, assessed outcomes, and main results. During the data extraction process, the authors identified substantial heterogeneity and complexity in microbiota-related outcomes across studies. Therefore, an additional table (Table 2) was developed to specifically synthesize gut microbiota findings, allowing a more structured and transparent presentation of these results beyond the main summary table. Data were extracted by the two reviewers (M.A.S.N. and R.S.P.) using a standardized form in Google Sheets, web-based spreadsheet software, Google LLC, with the variables of interest (Tables 3 and 2).

**Table 2:** Key microbial outcomes in studies reporting gut microbiota

Author, year	Kefir type and dose	Groups	Bacterial taxa	Microbiota assessment methodology	Main outcomes
Anwar, M.M. et al., [6]	Lyophilized milk kefir 150 mg/kg or L-MKG/SNESNS 100 mg/kg; Oral gavage	**G1**: control **G2**: Acute-IBD‐like model **G3**: Chronic-IBD‐like model **G4**: Chronic-IBD‐like model + L‐MKG **G5**: Chronic-IBD‐like model + L‐MKG/SNESNS	*Escherichia coli*,* Streptococcus mitis*,* Lactobacillus casei*,* Bacteroides fragilis*,* Salmonella typhi*,* Bifidobacterium dentium*,* Campylobacter fetus*,* Bacteroides vulgatus*,* Klebsiella aerogenes*,* Enterobacter cloacae*	Culture-based luminal bacterial count (CFU/mL) with phenotypic identification (API20E)	**G4 and G5 vs. G2 and G3**: Increased counts of *Escherichia coli*; Reduced counts of *S. typhi*,* B. dentium*,* C. fetu*s; **G5 vs. G2**,** G3 and G4**: Increased counts of *Streptococcus mitis*,* L. casei*,* B. fragilis;* **G4 and G5 vs. G3**: Reduced counts of *B. vulgatus*,* K. aerogenes*,* E. cloacae*. Overall, the treatment (especially in G5) was associated with restoration of bacterial counts of species considered beneficial to levels comparable to the control group and with lower counts of species considered harmful relative to the DSS-induced model, suggesting a modulatory effect on fecal bacterial populations, without reported statistical comparisons.
Erol and Özmen, [10]	Milk kefir, Water kefir, or Kombucha (0.2 mL/day); Oral gavage	**G1**: Control **G2**: 5% AA colitis + Milk kefir **G3**: 5% AA colitis + Water kefir **G4**: 5% AA colitis + Kombucha	Total aerobic mesophilic bacteria, *Lactobacillus spp.*,* Lactococcus spp.*, coliform bacteria	Culture-based fecal microbial analysis (media-dependent assay; CFU counts)	All groups: total aerobic mesophilic counts were approximately 7 log10 CFU at baseline (D0) in all groups; at D14, the highest TAMC values were observed in the G2. *Lactobacillus* spp. counts increased over the experimental period compared with baseline and reached approximately 8 log10 CFU at D14. *Lactococcus* spp. counts were approximately 7 log10 CFU at baseline, reaching approximately 8 log10 CFU at D14. Across groups: coliform counts varied throughout the study period; the highest coliform counts were observed in the G2, whereas the lowest counts were observed in the G4. Overall: the fermented beverage interventions were associated with temporal variations in fecal bacterial populations, characterized by increases in lactic acid bacteria over time and differences in coliform counts across groups, without reported statistical comparisons.
Wang et al., [16]	*Lactobacillus kefiranofaciens M1* encapsulated with sodium alginate/gellan gum/skim milk powder (10⁸ CFU/mouse/day), intragastrically gavage	**G1**: Normal control **G2**: DSS control **G3**: DSS + encapsulated *Lactobacillus kefiranofaciens M1* **G4**: DSS + encapsulated control (capsule without *Lactobacillus kefiranofaciens M1*)	*Lactobacillus spp.; Escherichia coli* (coliforms)	Culture-based fecal microbial analysis (CFU counts)	G3 showed a restoration trend of the lactobacilli/coliform ratio toward normal values, without statistical difference compared with G2.
Ye et al., [17]	Water kefir microbiota (Low dose: 4 × 10⁷, Middle dose: 2 × 10⁸, or High dose: 1 × 10⁹ CFU/day); Oral gavage	**Prevention groups**: (Treatment lasted 14 days, with DSS administration beginning on day 8) **G1**: Normal control **G2**: PBS + 3% DSS colitis **G3**:Low-dose kefir + DSS **G4**: Middle-dose kefir + DSS **G5**: High-dose kefir + DSS **Treatment groups**: (DSS was administered for 14 days, with treatment initiated on day 8) **G6**: DSS + PBS **G7**: DSS+ High-dose kefir	*Proteobacteria*,* Firmicutes; Lactobacillus*,* Escherichia_Shigella*,* unclassified_Enterobacteriaceae*	16 S rRNA gene sequencing (V3–V4 region) using Illumina MiSeq platform; OTU-based analysis	**G2 vs. G1**: ↑ *Proteobacteria*,* ↑ Escherichia_Shigella*,* ↑ unclassified_Enterobacteriaceae;* and ↓ *Firmicutes*,* ↓ Lactobacillus*; **G5 vs. G2**: ↓ *Proteobacteria; ↓ Escherichia_Shigella; ↓ unclassified_Enterobacteriaceae*. Overall, high-dose water kefir microbiota attenuated DSS-induced gut dysbiosis.
Youn et al., [18]	*Kluyveromyces marxianus* A4 (1 × 10⁸ CFU/mL) alone or combined with sulfasalazine (50 mg/kg/day), gavage	**G1**: Normal control **G2**: DSS control **G3**: DSS + Sulfasalazine **G4**: DSS + KMA4 **G5**: DSS + Sulfasalazine + KMA4 **G6**: DSS + Sb MYA-796 **G7**: DSS + Sb MYA-796 + Sulfasalazine	*Bacteroidota*,* Proteobacteria*,* Bacteroidaceae*,* Rikenellaceae*,* Oscillospiraceae*,* Lachnospiraceae*,* Bacteroides*,* Alistipes*,* Mucispirillum*,* Anaeroplasma*,* Colidextribacter*	16 S rRNA gene sequencing (V3–V4 region); beta-diversity analysis using weighted and unweighted UniFrac PCoA; taxonomic profiling; LEfSe (LDA > 2.0)	**G2 vs. G1**: DSS induced marked gut microbiota dysbiosis, with clear separation from G1 in both weighted and unweighted UniFrac PCoA analyses; **G5 vs. G2**: A4 + S shifted microbial community structure toward G1 in both weighted and unweighted UniFrac PCoA.; ↑ abundance of *Bacteroidota*,* Bacteroides*,* Bacteroidales*; and ↓ abundance of *Firmicutes*, *Clostridia* and *Oscillospiraceae*. Overall, kefir yeast plus sulfasalazine modulated gut microbiota toward a profile associated with reduced inflammation.
Zeng et al., [19]	*Saccharomyces cerevisiae* JKSP39 (10⁸ CFU/mouse/day) orally gavage for 3 weeks, isolated from Tibetan kefir grains	**G1**: Normal control **G2**: *F. nucleatum* + DSS **G3**: *F. nucleatum* + DSS + *Saccharomyces cerevisiae* JKSP39	*Bacteroides*,* Bifidobacterium*,* Akkermansia*,* Dubosiella*,* Turicibacter*,* Blautia*,* Rikenellaceae_RC9_gut_group*	Fecal 16 S rRNA gene sequencing (V3–V4 region, Illumina MiSeq PE300); OTU-based analysis; α-diversity (Chao1, Shannon); β-diversity (PCoA, Bray–Curtis, ANOSIM); taxonomic annotation by Naive Bayes classifier	**G2 vs. G1**: ↓ α-diversity and altered gut microbiota composition; ↑ relative abundance of *Bacteroides*,* Akkermansia*, *Rikenellaceae_RC9_gut_group*, and ↓ *Bifidobacterium* and *Dubosiella*; **G3 vs. G2**: ↑ Blautia. Overall, *Saccharomyces cerevisiae* JKSP39 altered gut microbiota composition and specific genera associated with inflammation without restoring overall microbial richness and diversity.
Zeng et al., [20]	Kefir supernatant, 0.2 mL/mouse/day orally for 3 weeks	**G1**: Normal control **G2**: *Fusobacterium nucleatum* control **G3**: DSS control **G4**: *Fusobacterium nucleatum* + DSS control **G5**: *Fusobacterium nucleatum* + DSS + Kefir supernatant	*Bacteroides*,* Parasutterella*,* Odoribacter*,* Blautia*,* Akkermansia*,* Lactobacillus*,* Helicobacter*,* Morganella*,* UBA1819*	Fecal 16 S rRNA gene sequencing (V3–V4 region, Illumina MiSeq); ASV-based analysis using QIIME2 with Silva database; β-diversity by PCoA (weighted UniFrac); group separation assessed by ANOSIM (Bray–Curtis); differential taxa identified by one-way ANOVA and LEfSe	**G4 vs. G1**: ↑ relative abundance of *Bacteroides* and *Parasutterella*; **G5 vs. G2**: ↑ *UBA1819*, ↑ *Morganella* and ↑ *Akkermansia*; **G5 vs. G3**: ↑ *Blautia*,* ↑ UBA1819*,* ↑ Morganella* and *↑ Akkermansia*; **G5 vs. G4**: ↓ *Bacteroides* and ↓ *Odoribacter*, and ↑ *Blautia* and ↑ *Akkermansia*; β-diversity ANOSIM analysis showed significant differences in microbial community structure between groups
Zhao et al., [21]	*Lactobacillus helveticus LZ-R-5*, 0.2 mL/mouse/day of 10⁸ or 10⁹ CFU (high and low dose), gavage	**G1**: Normal control **G2**: 3,5% DSS control **G3**: DSS + LHLZ-R-5 low dose **G4**: DSS + LHLZ-R-5 high dose	*Bacteroides*,* Erysipelatoclostridium*,* Muribaculaceae*,* Lachnospiraceae*,* Rikenellaceae*,* Oscillospiraceae*,* Clostridium leptum*,* Roseburia spp.*	Fecal 16 S rRNA gene sequencing (V3–V4 region, Illumina MiSeq); OTU analysis,; taxonomic assignment using RDP classifier with Silva database; α-diversity (Shannon); β-diversity (PCA, PCoA, Bray–Curtis); differential taxa by Kruskal–Wallis test with FDR correction and LEfSe	G2 vs. G1: ↓ α-diversity; ↑ *Bacteroidaceae* and ↓ *Muribaculaceae*,* Lachnospiraceae*,* Rikenellaceae* and *Oscillospiraceae*; G4 vs. G2: ↓ relative abundance of *Erysipelatoclostridium* and partial preservation of *Clostridium leptum*; no statistically significant restoration of overall α-diversity with R-5 treatment. Overall, R-5 partially corrected DSS-induced dysbiosis by modulating inflammation-associated taxa without fully restoring microbial diversity.

↓ indicates a reduction, ↑ indicates an increase in the score. CFU: Colony Forming Units; DSS: Dextran Sulfate Sodium; G1: Group 1; G2: Group 2; G3: Group 3; G4: Group 4; G5: Group 5; G6: Group 6; G7: Group 7; IBD: Inflammatory Bowel Disease; KMA4: *Kluyveromyces marxianus* A4; LHLZ-R-5: *Lactobacillus helveticus* LZ-R-5; L-MKG: lyophilized milk kefir grains; NC: Normal control; PBS: phosphate-buffered saline; Sb MYA-796: *Saccharomyces boulardii* ATCC MYA-796; SNESNS: Self‐nanoemulsifying self‐nanosuspension; TNBS: trinitrobenzene sulfonic acid

**Table 3:** Characteristics of studies assessing the effects of Kefir and its by-products on inflammation, oxidative stress, gut health, and immunity

Author (year)	Study design and duration	Kefir type and dose	Sample characteristics	Groups	Assessed outcomes	Main outcomes
Anwar, M.M. et al. [6]	Experimental study; 26-day total protocol (16-day induction DSS + 10-day treatment/intervention)	Lyophilized milk kefir 150 mg/kg or L-MKG/SNESNS 100 mg/kg; Oral gavage	*N* = 30 Male Albino rats (180 to 200 g) Age: 8 weeks old 5 groups (6 animals/group)	**G1**: Control **G2**: Acute-IBD‐like model **G3**: Chronic-IBD‐like model **G4**: Chronic-IBD‐like model + L‐MKG **G5**: Chronic-IBD‐like model + L‐MKG/SNESNS	Body weight, colon length, DAI, histopathology, behavioral tests, microbiota composition, cytokines (IL-1β, TNF-α, IL-6, IL-10), oxidative stress markers (MDA, SOD, NO, GST), neurotransmitters	G5 vs. G3 and G4: ↓ DAI, Body weight loss, Harmful bacteria (*S. typhi*,* K. aerogenes*,* E. cloacae*), inflammatory cytokines on brain, colon and serum **(IL-1β**,** TNF-α**,** IL-6)**, oxidative stress (MDA, NO); ↑ Colon length, anti-inflammatory cytokine **IL-10**, antioxidants on brain, colon and intestine (GST, SOD), Cognitive function, Neurotransmitter levels, Beneficial bacteria (*E. coli*,* L. casei*,* B. fragilis*,* S. mitis*)
Chen et al. [7]	Experimental study + In vitro (14 days of treatment)	*Lactobacillus kefiranofaciens* M1, 10⁷ or 10⁸ CFU/day; Oral gavage	*N* = 40 Female C57BL and TLR2 knockout Age: 8–10 weeks old 5 groups (8 animals/ group)	**G1**: PBS control **G2**: DSS colitis control **G3**: DSS + 10⁷ CFU/day *L. kefiranofaciens* M1 **G4**: DSS + 10⁸ CFU/day *L. kefiranofaciens* M1 **G5**: TLR2 Knockout + DSS + M1 (10⁸ CFU/day)	Bleeding score, colon length, histology, cytokines (IL-1β, TNF-α, IL-10)	G3 and G4 vs. G2: ↓ Rectal bleeding score (significant at 10⁸ CFU/day), Inflammatory score, **IL-1β and TNF-α** in colon cultures, ↑ Colon length, **IL-10** in colon cultures
Deeseenthum et al., [8]	Experimental animal study; 17 days total (acclimatized for 7 days + 10-day treatment post-colitis induction with TNBS)	Hawm Nil rice milk kefir powder (HNKP), 150 mg/kg/day; Cow milk kefir powder (CMKP), 150 mg/kg/day; Prednisolone, 5 mg/kg/day; orally	*N* = 42 male Wistar rats (180–200 g) 7 groups (6 animals/group) Random allocation	**G1**: PBS control **G2**: HNKP control **G3**: CMKP control **G4**: TNBS colitis + PBS **G5**: TNBS colitis + HNKP **G6**: TNBS colitis + CNKP **G7**: TNBS colitis + Prednisolone	Nitric oxide (NO), lipid peroxidation (TBARS/MDA), and superoxide dismutase (SOD) activity	G5, G6 and G7 vs. G4: ↓ MDA/TBARS, ↓NO, ↑ SOD activity; effects comparable to prednisolone; considered safe and potentially beneficial as a functional food supplement
Deeseenthum; Luang-In; Chunchom, [9]	Experimental animal study; 17 days (acclimatized for 7 days + 10-day treatment post-colitis induction with TNBS)	Hawm Nil rice milk kefir powder (HNKP), 150 mg/kg/day; Cow milk kefir powder (CMKP), 150 mg/kg/day; Prednisolone, 5 mg/kg/day, orally	*N* = 42 male Wistar rats (180–200 g) 7 groups (6 animals/group) Random allocation	**G1**: PBS control **G2**: HNKP control **G3**: CMKP control **G4**: TNBS colitis + PBS **G5**: TNBS colitis + HNKP **G6**: TNBS colitis + CNKP **G7**: TNBS colitis + Prednisolone	TNF-α levels, hematological and biochemical parameters (RBC, WBC, Hct, Hb, MCV, MCH, MCHC, platelets, neutrophils, lymphocytes, total protein, blood sugar, blood urea nitrogen, creatinine, uric acid, albumin, globulin, total bilirubin, cholesterol, TG, HDL, LDL, ALT, ALP, AST)	G5, G6 and G7 vs. G4: **↓ TNF-α levels;** ↓ ALT; results comparable to prednisolone; no adverse effects observed
Erol and Özmen, [10]	Experimental study, 14 days total (7 before + 7 after induction)	Milk kefir, Water kefir, or Kombucha (0.2 mL/day); Oral gavage	*N* = 40 Male CD-1 mice (30–35 g) Age: 6–8 weeks old 4 groups (10 animals/group)	**G1**: Control **G2**: 5% AA colitis + Milk kefir **G3**: 5% AA colitis + Water kefir **G4**: 5% AA colitis + Kombucha	Colon histopathology, fecal microbial (coliforms, *Lactobacillus spp.*), and beverage properties	G2 and G3 vs. G1: ↓ Histological lesion severity G3: ↓ Inflammatory scores (mild), coliform counts slightly, ↑ *Lactobacillus* and *Lactococcus* counts G4:↓ Histopathology score and coliforms
Nascimento da Silva et al., [11]	Experimental study, 9 days total	Fermented milk kefir (ad libitum, ~ 25 mL/day)	*N* = 32 Male Wistar rats (180–250 g) Age: 8–10 weeks old 4 groups (8 animals/group)	**G1**: Baseline control **G2**: Kefir control **G3**: 5% DSS colitis **G4**: 5% DSS colitis + milk kefir	DAI, histology, ultrastructure, goblet cells, SCFAs	G4 vs. G3: ↓ DAI, histopathological score, neutrophil infiltration and edema, ↑ preservation of epithelial architecture and TJ, autophagosomes, slight ↑ in acetate and propionate levels, ↑ trend in goblet cell index
Senol et al., [12]	Experimental study; 14 days total	Milk kefir (5 mL/day); Orogastric gavage	*N* = 24 Male Wistar albino rats (198–264 g) Age: 18 weeks old 6 per group/4 groups	**G1**: Normal control **G2**: Kefir control **G3**: 3% DSS colitis **G4**: 3% DSS colitis + milk kefir	Body weight, DAI (diarrhea, stool blood), colon length/weight, histology, MPO, TNF-α, IL-10, MDA, iNOS	G4 vs. G3: ↓ DAI on days 3 and 5, microscopic colitis score, **TNF-α levels**, MPO, iNOS, ↑ Colon length, ↔ **IL-10** and MDA levels G2 vs. G3: ↓ Inflammation severity (milder inflammatory infiltrate and submucosal edema in G2 vs. extensive ulceration in G3)
Seo et al., [13]	Experimental study + in vitro; 10 days of treatment (in vivo)	EV from *Lactobacillus kefir*,* L. kefiranofaciens*,* L. kefirgranum* (3 × 10⁸ or 3 × 10¹⁰ particles/day); Oral administration	*N* = 25 Male Balb/cAnNCrljOri mice Age: 8 weeks old 5 groups (5 animals/group)	**G1**: Normal control **G2**: TNBS colitis + vehicle **G3**: TNBS + EV low dose **G4**: TNBS + EV high dose **G5**: TNBS + prednisolone	Body weight, rectal bleeding, stool consistency, colon histology, plasma MPO	G3 and G4 vs. G2:↓ Body weight loss, rectal bleeding, leukocyte infiltration, MPO,↑ Stool consistency, goblet cells
Sevencan et al., [14]	Experimental study, 14 days total	Milk kefir, 10% or 30% in PBS (~ 10 or 30 ml/day); Oral administration	*N* = 54 Male Wistar Albino rats (200–250 g) Age: 18 weeks old 8 per group/3 groups (controls), 10 per group/3 groups (colitis groups)	**G1**: Normal control **G2**: Kefir 10 Control **G3**: Kefir 30 control **G4**: Colitis control **G5**: Kefir 10 colitis **G6**: Kefir 30 colitis	Diarrhea, bloody stools, colon weight/length, macroscopic damage, histopathology, fluid/chow intake, body weight	G5 vs. G4 and G6:↓ Bloody stool and diarrhea, Colon weight/length ratio, macroscopic damage score, ↔ Microscopic damage score
Spinler et al., [15]	Experimental animal study; 14 days total (9 days intervention)	Lifeway^®^ kefir, 200 mcL by gavage 2x or 3x/day; or PBS 200 mcL by gavage 2x/ day	*N* = 20 Female mice C57BL/6 Age: 8 weeks old 4 groups (5 animals/group)	**G1**: PBS 2x daily + *Clostridium difficile* **G2**: Kefir 2x daily + *Clostridium difficile* **G3**: Kefir 3x daily + *Clostridium difficile* **G4**: Kefir 3x daily control	Body weight, clinical score, survival, *Clostridium difficile* load (qPCR), toxin A/B levels (ELISA)	G3 ↑ disease severity; ↓ body weight; compared with the other groups - all animals moribund by day 3; G2 milder disease; no protection from kefir; increased severity not explained by higher bacterial load or toxins; potential host immune susceptibility
Wang et al., [16]	Experimental animal study; 18 days total (18 days treatment with seven final days DSS-induced colitis)	*Lactobacillus kefiranofaciens M1* encapsulated with sodium alginate/gellan gum/skim milk powder (10⁸ CFU/mouse/day), intragastrically gavage	*N* = 32 female mice C57BL/6 Age: 8–10 weeks old 4 groups (8 animals/group)	**G1**: Normal control **G2**: DSS colitis control **G3**: DSS + encapsulated *Lactobacillus kefiranofaciens M1* **G4**: DSS + encapsulated control (capsule without *Lactobacillus kefiranofaciens M1*)	Colon length, rectal bleeding score, histological score, faecal *Lactobacilli vs. E. coli* counts, M1 viability (CFU) after storage, heating, SGFT, and bile salt exposure	Encapsulated M1 maintained viability under heat/storage/gastrointestinal stress; G3 vs. G2 and G4: ↓ DSS-induced rectal bleeding and ↓ inflammation score; improved *Lactobacillus*/coliform ratio; effect not seen in G4 with capsule alone—confirming that benefit was from M1, not the matrix
Ye et al., [17]	Experimental study, 14 days total	Water kefir microbiota (Low dose: 4 × 10⁷, Middle dose: 2 × 10⁸, or High dose: 1 × 10⁹ CFU/day); Oral gavage	*N* = 70 Female C57BL/6J mice (18–21 g) Age: 8 weeks old 10 per group/ 5 groups (intervention), 10 per group/2 groups (treatment)	**Prevention groups**: (Treatment lasted 14 days, with DSS administration beginning on day 8) **G1**: Normal control **G2**: PBS + 3% DSS colitis **G3**:Low-dose kefir + DSS **G4**: Middle-dose kefir + DSS **G5**: High-dose kefir + DSS **Treatment groups**: (DSS was administered for 14 days, with treatment initiated on day 8) **G6**: DSS + PBS **G7**: DSS+ High-dose kefir	DAI, colon length, spleen index, cytokines, TJ proteins, microbiota composition	G5: ↓ DAI, spleen index, body weight loss, histological damage, **IL-1β**,** IL-6**,** TNF-α**, Cox-2, iNOS, TLR4/MyD88/NF-κB activation, ↑ colon length, expression of TJ proteins (ZO-1, claudin-1, occludin), **IL-10**, beneficial microbiota balance; G3, G4 and Treatment groups: minimal effects.
Youn et al., [18]	Experimental animal study; 3 weeks (acclimatized for 7 days + DSS-induced *ad libitum* 3% w/v for 7 days + 7-day treatment post-induction)	*Kluyveromyces marxianus* A4 (1 × 10⁸ CFU/mL) alone or combined with sulfasalazine (50 mg/kg/day), gavage	*N* = 56 male mice BALB/c Age: 6 weeks old 7 groups (8 animals/group)	**G1**: Normal control **G2**: 3% DSS colitis control **G3**: DSS + sulfasalazine **G4**: DSS + KMA4 **G5**: DSS + sulfasalazine + KMA4 **G6**: DSS + Sb MYA-796 **G7**: DSS + Sb MYA-796 + sulfasalazine	Body weight, colon length, spleen/liver weight, hematological parameters (RBC, HGB, HCT, MCV, MCH, MCHC, RDW, MPV, PLT, WBC, neutrophils, lymphocytes, MONO, EOS, BASO), histological colonic score, gene expression via qRT-PCR (IL-1β, IL-6, TNF-α, IL-10, IL-4, IL-12p40, Ifn-γ, Cox-2, inos, ZO-1, Occludin, Claudin-1); gut microbiota composition (16 S rRNA sequencing); β-glucan content	G3 and G4 showed moderate improvements vs. G2; G5 showed strongest anti-inflammatory effect: ↑ body weight, ↑ colon length, **↓ IL-6**,** ↓ TNF-α**,** ↑ IL-10**, ↑ occludin, ↓ histological score, ↑ *Bacteroidota* abundance vs. G2; G6 and G7 showed similar but milder effects than G3 and G4; so G5: The combination KMA4 + S showed synergistic potential in alleviating DSS-induced colitis through immune modulation and gut barrier support
Zeng et al., [19]	Experimental animal study; 4 weeks total (3 weeks of treatment after 1-week acclimatization)	*Saccharomyces cerevisiae* JKSP39 (10⁸ CFU/mouse/day) orally gavage for 3 weeks, isolated from Tibetan kefir grains	*N* = 30 male mice C57BL/6J Age: 6 weeks old 3 groups (10 animals/group): housed under controlled conditions and given DSS to induce colitis and *F. nucleatum* to promote inflammation	**G1**: Normal control **G2**: *F. nucleatum* + DSS colitis **G3**: *F. nucleatum* + DSS + *Saccharomyces cerevisiae* JKSP39	DAI; body weight; colon length; histology (goblet cells); gene expression via qRT-PCR of colon tissue; cytokines (TNF-α, IL-6, IL-17 F, IL-4, IL-10), ROS markers (MPO, SOD, CAT, MDA, H₂O₂), barrier proteins (MUC2, Claudin-1, Occludin, ZO-1), ER stress (CARD3, chk2, NF-κB, JNK, Bcl-2/Bax), gut microbiota composition (16 S rRNA)	G3 vs. G1 and G2: ↑ body weight, ↓ DAI, restored colon length; ↓ proinflammatory cytokines **(TNF-α**,** IL-6**,** and IL-17 F**), ↑ anti-inflammatory cytokines **(IL-4);** ↓ ROS (MPO, MDA, H₂O₂), ↓ ER stress ( ↓ NF-κB, JNK and ↑ Bcl-2/ Bax ratio); modulated gut microbiota (↑ *Blautia*, ↓ *Turibacter*, ↓ *Rikenellaceae_RC9_gut_group*); improved gut barrier by ↑ TJ proteins (MUC2, Claudin-1, ZO-1, occludin) and ↑ number of goblet cells per crypt
Zeng et al., [20]	Experimental animal study; 4 weeks total (3 weeks of treatment after 1-week acclimatization)	Kefir supernatant, 0.2 mL/mouse/day orally for 3 weeks	*N* = 50 male mice C57BL/6J mice Age: 6 weeks old Randomly divided into 5 groups (10 animals/group)	**G1**: Normal control **G2**: *Fusobacterium nucleatum* control **G3**: DSS colitis control **G4**: *Fusobacterium nucleatum* + DSS **G5**: *Fusobacterium nucleatum* + DSS + Kefir supernatant	Inflammatory markers (IL-6, IL-17 F, TNF-α, IL-4, IL-10), oxidative stress (T-SOD, CAT, MPO, MDA), colon histology, epithelial barrier integrity (MUC2, claudin-1, occludin), fecal gut microbiota (16 S rRNA), fecal metabolomics (L-arginine pathway)	G5 vs. G4: reduced inflammation (**↓TNF-α**,** IL-6**,** IL-17 F; ↑IL-4**,** IL-10)**, oxidative stress (↓MPO, CAT, MDA), and improved epithelial barrier (↑MUC2, claudin-1, occludin); mitigated gut microbiota dysbiosis by ↑ *Akkermansia* and *Blautia* and ↓ *Bacteroides*; modulated L-arginine biosynthesis pathway
Zhao et al., [21]	Experimental animal study; 3 weeks (1 week acclimatization + 2 weeks treatment)	*Lactobacillus helveticus LZ-R-5*, 0.2 mL/mouse/day of 10⁸ or 10⁹ CFU (high and low dose), gavage	*N* = 40 male mice BALB/c mice Age: 6 weeks old Randomly divided into 4 groups (10 animals/group)	**G1**: Normal control **G2**: 3,5% DSS colitis control **G3**: DSS + LHLZ-R-5 high dose **G4**: DSS + LHLZ-R-5 low dose	DAI, fecal SCFA concentrations, qPCR, body weight, colon length, histology (colon, ileum, liver, thymus, spleen), inflammatory markers (MPO, IL-6, IL-10, TNF-α, IL-1β), biomarkers of liver or kidney dysfunction (AST, ALT, BUN, creatinine) gut barrier markers (MUC2, ZO-1, Claudin-1, Occludin), gut microbiota (16 S rRNA), functional metagenomics (KEGG), correlation with inflammation markers	G3 showed ↓ DAI, ↑ SCFAs (especially butyrate), ↓ pro-inflammatory cytokines (**IL-6**,** TNF-α**), ↑ intestinal barrier genes (MUC2, Claudin-1, ZO-1), partially restored gut microbiota (↓ *Bacteroides*, *Erysipelatoclostridium*; ↑ *Lachnospiraceae*), ↑ body weight/colon length; alleviated liver and spleen immune stress without toxicity; IL-10 response minimal. G4 showed intermediate effects

↔ indicates no difference, ↓ indicates a reduction, ↑ indicates an increase in the score. AA: acetic acid; ALP: alkaline phosphatase; ALT: alanine aminotransferase; AST: aspartate aminotransferase; BALB: bagg albino; Bax: Bcl-2-Associated X; Bcl-2: B-cell lymphoma 2; CAT: catalase; CFU: colony forming units; CMKP: cow milk kefir powder; Cox-2: cyclooxygenase-2; DAI: Disease Activity Index; DPPH: 2,2-diphenyl-1-picrylhydrazyl hydrate; DSS: Dextran Sulfate Sodium; ELISA: Enzyme-Linked Immunosorbent Assay; ER: endoplasmic reticulum; EV: extracellular vesicles; FRAP: ferric reducing antioxidant activity; G1: group 1; G2: group 2; G3: group 3; G4: group 4; G5: group 5; G6: group 6; G7: group 7; Hb: hemoglobin; Hct: hematocrit; HDL: high density lipoprotein; HNKP: Hawm Nil rice kefir powder; IBD: inflammatory bowel disease; Ifn-γ: interferon-γ; IL: interleucin; IL-1β: interleukin-1 beta; IL-6: interleukin-6; IL-10: interleukin-10; iNOS: inducible nitric oxide synthase; JNK: C-jun N-terminal kinase; KEGG: Kyoto Encyclopedia of Genes and Genomes; KMA4: *Kluyveromyces marxianus* A4; LDL: low density lipoprotein; LHLZ-R-5: *Lactobacillus helveticus* LZ-R-5; L-MKG: lyophilized milk kefir grains; MCH: mean corpuscular hemoglobin; MCHC: mean corpuscular hemoglobin concentration; MCV: mean corpuscular volume; MDA: malondialdehyde; MPO: myeloperoxidase; MUC2: mucin 2; MyD88: myeloid differentiation primary response 88; NC: normal control; NF-κB: nuclear factor kappa beta; PBS: phosphate-buffered saline; qRT-PCR: real time quantitative polymerase chain reaction; RBC: red blood cell; rRNA: ribosomal ribonucleic acid; Sb MYA-796: *Saccharomyces boulardii* ATCC MYA-796; SFCAs: short-chain fatty acids; SGFT: simulated gastrointestinal fluid test; SNESNS: self‐nanoemulsifying self‐nanosuspension; SOD: superoxide dismutase; TBARS: thiobarbituric acid reactive substances; TG: triglycerides; TJ: tight junction; TLR: toll-like receptor; TNBS: trinitrobenzene sulfonic acid; TNF-α: tumor necrosis factor alpha; WBC: white blood cell; ZO-1: zonula occludens-1

### Assessment of Risk of Bias

The risk of bias of the included studies was assessed using the SYRCLE’s Risk of Bias tool [22], independently and in parallel by two authors (M.A.S.N. and R.S.P.). Each domain was evaluated individually and classified as low, high, or unclear risk of bias according to the tool’s criteria. The risk of bias was considered qualitatively when interpreting the findings of the included studies.

## Results

###  Selection and Characteristics of the Studies

**Fig. 1:**
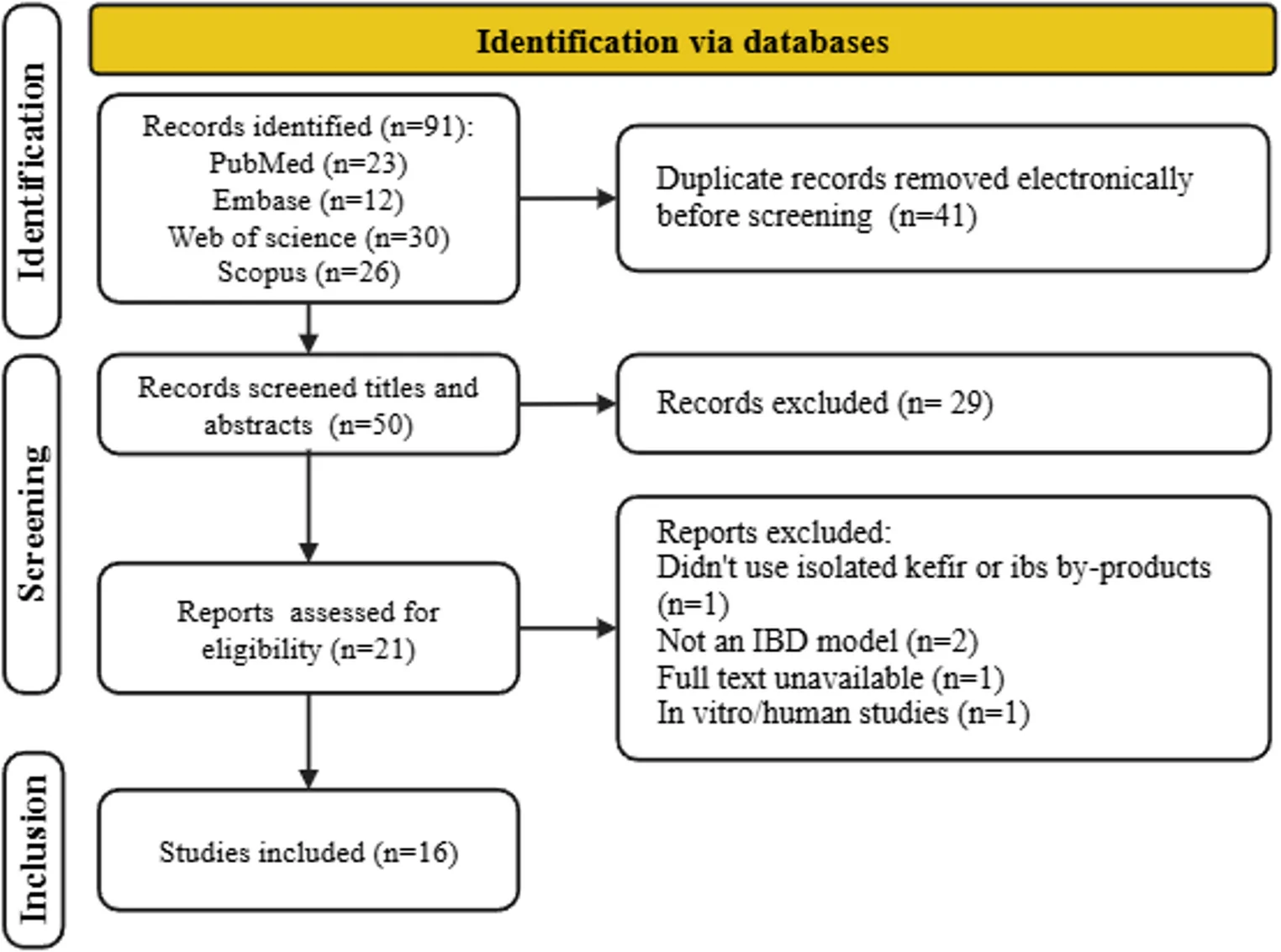
PRISMA flow diagram

A Total of 91 citations were retrieved from the PubMed/MEDLINE, Web of Science, Embase, and Scopus databases as of May 2025. Of the citations retrieved from the databases, 50 titles remained after duplicates were removed using the Rayyan QCRY software. During the screening of titles and abstracts, 29 records were excluded according to the initial exclusion criteria. Of the remaining 21 articles, 15 were unanimously included by the first two reviewers (M.A.S.N. and R.S.P.). The other six presented disagreements, which were resolved by a third reviewer (A.C.P.), who decided to exclude four and include two. However, we are unable to access one of the included studies, resulting in its exclusion. Therefore, this systematic review included 16 articles of experimental studies with mice/ rats (Fig. 1).

The population consisted of 585 rats and mice of the following species/lineages: Albino, C57BL, TLR2 knockout, Wistar, CD-1, and BALB/c; aged 6–18 weeks, mainly male (72,3%; *n* = 423). The substances used in the interventions were milk kefir (50%; *n* = 8), microorganisms isolated from kefir (43,75%, *n* = 7), rice milk kefir (12,5%; *n* = 2), water kefir (6,25%; *n* = 1), milk kefir supernatant (6,25%; *n* = 1). All studies had follow-up periods ranging from 9 to 28 days, and the substances were administered orally by gavage or *ad libitum.*

The most studied characteristics were the animals’ body weight, colon length or weight, microbiota composition, inflammatory cytokines, oxidative stress markers, and disease activity index (DAI).

### Evaluated Outcomes

#### Inflammatory, Immunological and Molecular Biomarkers

Kefir consumption and its by-products demonstrated consistent modulatory effects on inflammatory, immunological, and molecular biomarkers in experimental colitis models. Across the included studies, reductions in classical pro-inflammatory cytokines were frequently observed. Tumor necrosis factor-alpha (TNF-α) was reduced in seven studies, encompassing the total dataset, regardless of kefir source, supporting its role as a consistent target of kefir-based interventions [7, 12, 17–21]. Similarly, interleukin-1 beta (IL-1β) and interleukin-6 (IL-6), cytokines commonly elevated in colitis, were suppressed in four and five studies, respectively, reinforcing the anti-inflammatory potential of kefir [7, 17–21].

Conversely, the anti-inflammatory cytokine interleukin-10 (IL-10) was increased in most studies (*n* = 5) that evaluated this outcome, indicating a convergent immunoregulatory response [7, 17–20]. Interleukin-4 (IL-4) was upregulated in only two studies, suggesting that this effect may be strain-specific or dependent on host-related factors such as animal species and strain, sex, microbiota composition or differences in colitis induction protocols, which were not consistently explored across experimental models [19, 20]. In addition to cytokines, inflammation-related enzymes were also modulated. Ye et al. [17] and Youn et al. [18] reported reduced expression of cyclooxygenase-2 (COX-2) and inducible nitric oxide synthase (iNOS), while decreased myeloperoxidase (MPO) activity was observed in four studies, further supporting attenuation of inflammatory activity [12, 17–21].

At the molecular and immunological level, kefir and kefir-derived microorganisms influenced key signaling pathways associated with immune regulation, cellular stress, and apoptosis. Chen et al. [7] demonstrated that *Lactobacillus kefiranofaciens* M1 exerted immunomodulatory effects through Toll-like receptor 2 (TLR2) signaling, while Youn et al. [18] reported immune-related benefits mediated via the TLR4/MyD88/NF-κB pathway, alongside reductions in inflammatory cytokines and iNOS expression [7, 18]. Furthermore, Zeng et al. [19] showed that kefir administration reduced endoplasmic reticulum stress by downregulating nuclear factor kappa B (NF-κB), c-Jun N-terminal kinase (JNK), and checkpoint kinase 2 (Chk2), while increasing the Bcl-2/Bax ratio, suggesting attenuation of apoptosis [19].

Broader immunological effects were also reported. Zhao et al. [21], using functional metagenomic analysis based on the Kyoto Encyclopedia of Genes and Genomes (KEGG), demonstrated improvements in immune-metabolic regulation and microbial balance. Consistently, Youn et al. [18] observed changes in immune cell profiles and cytokine expression, further reinforcing the immunomodulatory potential of kefir and its derivatives [18, 21].

#### Oxidative Stress Markers

Kefir intake and its derivatives have been shown to exhibit antioxidant properties by modulating oxidative stress markers. Reductions in malondialdehyde (MDA) levels were reported across different formulations in four studies, suggesting a general antioxidative effect of kefir, regardless of the model or preparation [9, 19–21]. Changes in antioxidant enzyme activity, including catalase (CAT) and superoxide dismutase (SOD), were also reported. However, as these enzymes are components of the endogenous antioxidant defense system, variations in their activity should be interpreted with caution. Decreases in CAT and SOD activity do not necessarily reflect reduced oxidative stress and may depend on factors such as oxidative burden, disease severity, and experimental conditions [19]– [20]. Notably, only Zeng et al. [19] reported a reduction in hydrogen peroxide (H_2_O_2_), which may be specific to the yeast strain or experimental model used [19].

#### Gut Health Markers

Studies included in the microbiota-specific summary table (Table 2) demonstrated that kefir and kefir-derived interventions modulated gut microbial composition in experimental colitis models, although the direction, magnitude, and breadth of these effects varied substantially across studies. Culture-based fecal analyses showed modulation of selected bacterial populations rather than global normalization. Specifically, Wang et al. [16] reported a restoration trend of the lactobacilli-to-coliform ratio following administration of encapsulated Lactobacillus kefiranofaciens M1, although this effect did not reach statistical significance when compared with the DSS control group [16]. Similarly, Anwar et al. [6] observed reduced counts of pathogenic bacteria, including Salmonella typhi, Klebsiella aerogenes, and Enterobacter cloacae, alongside increased counts of beneficial taxa such as Lactobacillus casei and Streptococcus mitis following L-MKG/SNESNS supplementation [6].

High-throughput 16 S rRNA gene sequencing studies consistently demonstrated significant alterations in gut microbiota structure, as evidenced by β-diversity analyses. Ye et al. [17] showed that high-dose water kefir attenuated DSS-induced dysbiosis, with shifts in dominant phyla and genera [17]. Youn et al. [18] further demonstrated that kefir yeast combined with sulfasalazine significantly shifted microbial community structure, as confirmed by weighted and unweighted UniFrac analyses, and altered abundance of Bacteroidota, Firmicutes, Clostridia, and Oscillospiraceae [18].

More detailed genus-level analyses were reported in studies using kefir-derived yeast or supernatant preparations. Zeng et al. [19] demonstrated that Saccharomyces cerevisiae JKSP39 significantly altered gut microbiota composition, characterized by reduced α-diversity indices (Chao1 and Shannon) in colitis models and increased relative abundance of Blautia, without full restoration of microbial richness or diversity [19]. In a subsequent study, Zeng et al. [20] reported that kefir supernatant supplementation significantly increased the relative abundance of Blautia and Akkermansia while reducing Odoribacter, with significant differences in microbial community structure between treatment groups [20]. Zhao et al. [21] similarly observed partial modulation of DSS-induced dysbiosis following Lactobacillus helveticus LZ-R-5 administration, with reduced Erysipelatoclostridium, without complete recovery of overall microbial diversity, in addition to reporting an increase in short-chain fatty acid levels, particularly butyrate [21].

Beyond microbial composition, markers of intestinal barrier integrity were evaluated in multiple studies included in Table 2. Ye et al. [17], Youn et al. [18], Zeng et al. [20], and Zhao et al. [21] consistently reported statistically significant upregulation of tight junction–related proteins, including ZO-1, claudin-1, occludin, and MUC2, following kefir or kefir-derived interventions, suggesting that improvements in epithelial barrier function may occur independently of full microbiota normalization [17, 18, 20, 21].

#### Clinical and Histological

Improvements in clinical parameters and histological architecture were consistently reported following kefir supplementation. Six studies reported reductions in the Disease Activity Index (DAI), histological damage, and body weight loss in models of colitis treated with kefir or its components, highlighting a robust protective effect across different formulations and experimental designs [10, 12, 17, 19–21]. Three other studies also observed recovery in colon length, reinforcing kefir’s role in supporting intestinal structural integrity [6, 18]. Similarly, four studies reported reductions in rectal bleeding and improved stool consistency, suggesting improvements in the clinical symptoms of colitis [7, 12–14].

Histological improvements, including the restoration of epithelial integrity and an increase in goblet cell counts, were observed in four studies, reinforcing kefir’s protective effects at the tissue level [11, 18–20]. Reductions in neutrophil infiltration and edema were also observed by Nascimento da Silva et al. [11]. In contrast, Ye et al. [17] reported an improvement in the spleen index, suggesting broader systemic benefits [11, 17]. In contrast to the predominantly positive outcomes, Spinler et al. [15], reported a worsening of colitis symptoms and weight loss with high-dose kefir administration, indicating that beneficial effects may be dose-dependent and formulation-specific [15].

### Kefir Types and Dosages

As seen, kefir consumption showed several effects among studies. Traditional milk kefir was associated with reductions in TNF-α, IL-1β, and IL-6. However, the administered dosage was not consistently reported across studies. Specifically, only Senol et al. [12] explicitly described the administration of 5 mL of kefir once daily for 14 days. In contrast, microbial fractions of water kefir administered at 2 × 10⁹ CFU/day were linked to reductions in TNF-α, IL-1β, and IL-6. Kefir-derived strains, such as *Lactobacillus kefiranofaciens* M1 and *Lactobacillus helveticus* LZ-R-5, both administered at 1 × 10⁹ CFU/day, promoted similar anti-inflammatory responses and increased IL-10 levels [7, 11, 12, 14, 17, 21].

These same interventions, particularly milk kefir and *L. helveticus* LZ-R were also associated with improved clinical and histological parameters, including reductions in Disease Activity Index, rectal bleeding, and histological damage, as well as recovery of colon length and preservation of epithelial structure [11, 12, 14, 21].

Regarding oxidative stress, antioxidant effects were observed with rice milk kefir powder at doses of 100–200 mg/kg, Saccharomyces cerevisiae JKSP39 at 1 × 10⁸ CFU/day, and Lactobacillus helveticus LZ-R-5. These interventions were consistently associated with reductions in MDA, while changes in SOD activity varied across studies, including increases indicative of restored antioxidant defense. A reduction in hydrogen peroxide H₂O₂ was reported only for rice milk kefir powder in Zeng et al. [8, 19, 21].

Improvements in gut microbial balance and intestinal barrier markers were found in studies using *L. helveticus* LZ-R-5, milk kefir supernatant at 200 µL/day, and water kefir microbiota, with increased abundance of beneficial genera and upregulation of tight junction proteins such as ZO-1, Claudin-1, and Occludin [17, 20, 21].

Finally, immunomodulatory and molecular effects were reported following treatment with *L. kefiranofaciens M1*, *Kluyveromyces marxianus* A4 at 1 × 10⁸ CFU/day, and *S. cerevisiae* JKSP39 [7, 18, 19].

### Results of Risk of Bias Assessment

The assessment of the risk of bias in the 16 studies included in this review, 10 (62.5%) were judged to have an unclear/moderate risk of bias, 5 (31.25%) had a high risk of bias, and only 1 study (6.25%) was considered to have a low risk of bias.

**Fig. 2:**
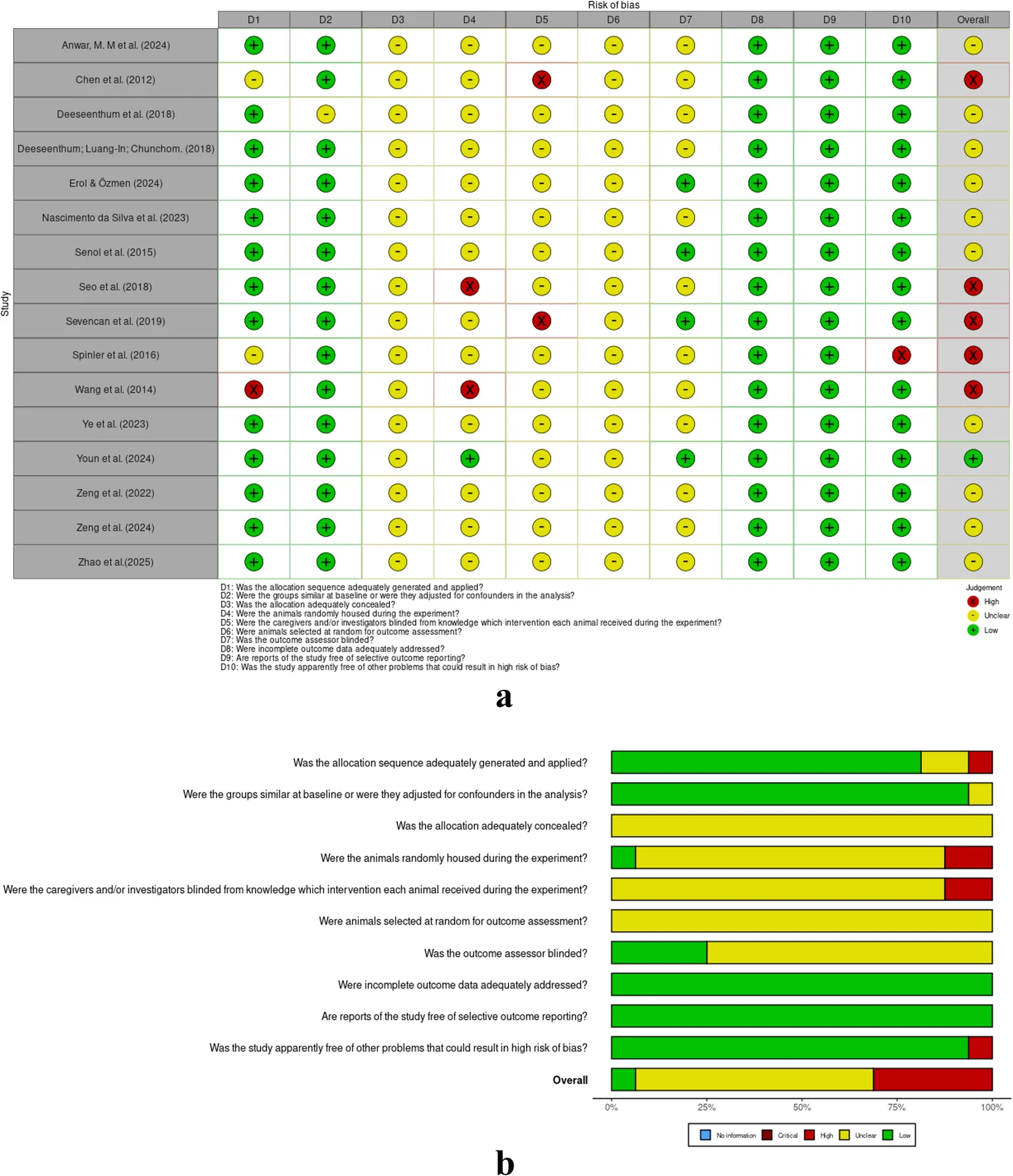
(**a**) Risk of bias represented by each item assessed and distribution of risk of bias across the studies included in the systematic review. (**b**) Distribution of risk of bias across the assessed domains for all included studies

The domains most frequently rated as “Unclear” were Q3 (allocation concealment), Q5 (blinding of participants and personnel), and Q6 (blinding of outcome assessors), indicating that information regarding randomization and blinding procedures was often insufficient or inadequately reported. This pattern was especially consistent across the studies classified as unclear overall (Fig. 2a).

In contrast, Q1 (random sequence generation) and Q9 (selective outcome reporting) were commonly rated as “Low” risk in most studies, reflecting more reliable reporting in these aspects (Fig. 2b).

### Limitations of the Study

This review presents certain limitations that warrant consideration. Notably, the studies included exhibited marked heterogeneity with respect to the type of kefir administered, which may influence biological effects and compromise comparability across findings. Furthermore, substantial variation in the dosages and intervention protocols was observed, hindering the ability to establish consistent dose–response relationships and limiting the strength of overall inferences. Collectively, these sources of heterogeneity constrain the generalizability of the conclusions and underscore the necessity for future research adopting standardized kefir formulations and dosing strategies to enhance methodological consistency and interpretability.

In addition, another important limitation of this review relates to the risk of bias identified in the included studies, as detailed in the previous section. A substantial proportion of the studies were classified as having an unclear or high risk of bias, largely due to insufficient reporting of key methodological domains, particularly allocation concealment and blinding of participants, personnel, and outcome assessors. Although random sequence generation and selective outcome reporting were generally described more adequately, the lack of transparency in other critical domains may compromise internal validity and weaken confidence in the reported effects. This limitation emphasizes the need for future experimental studies to report randomization and blinding procedures in a clear and comprehensive manner, thereby improving methodological quality and strengthening the evidence base in this field.

On the other hand, the review has strengths such as a comprehensive and methodologically structured search strategy, incorporating multiple reputable and extensive bibliographic databases to identify relevant literature. Sources such as PubMed/MEDLINE, Embase, Scopus, and Web of Science were systematically queried, ensuring broad coverage of published studies. The search strategy was carefully designed with well-defined keywords and controlled vocabulary terms. By leveraging these robust and established databases in conjunction with a detailed search framework, the review aimed to maximize retrieval sensitivity and enhance the overall rigor of the evidence-gathering process.

## Discussion

The findings of this study consistently suggest that kefir consumption reduces inflammation in IBD. Initially, this outcome was expected, but the analyses revealed a broader spectrum of actions, including antioxidant effects, restoration of epithelial integrity, modulation of the gut microbiota, and a consequent reduction in disease activity. The use of probiotics, prebiotics, and symbiotics as adjuvants in reducing IBD-related symptoms has been increasing, as evidenced by the rise in their commercialization and the growing number of publications on the subject [23–28]. In this context, kefir has been recognized as one of the most promising options.

The anti-inflammatory effects of kefir were consistent across the included studies, with a significant reduction in the expression of pro-inflammatory cytokines such as IL-1β, IL-6, IL-17 F, IFN-γ, and TNF-α, and an increase in anti-inflammatory cytokines like TGF-β, IL-10, and IL-4 [6, 7, 9, 12, 17–21]. These findings suggest that kefir has an immunomodulatory action, capable of rebalancing the exacerbated inflammatory response typical of IBD. These results align with previous studies that highlight the potential of kefir as an agent that reduces inflammation [29, 30]. Considering that the inflammatory environment of IBDs is the most important mechanism of these diseases and their symptoms [31, 32], an anti-inflammatory approach can be one of the most significant ways to improve the quality of life for these patients.

Another relevant aspect was the reduction of oxidative stress, evidenced by decreased levels of MDA, MPO, NO, and H₂O₂ [6, 8, 12, 13, 19–21] as well as increased activity of antioxidant enzymes such as SOD and CAT [6, 8, 20]. Oxidative stress is also closely linked to IBD progression through inflammatory responses, which increase the generation of reactive oxygen species and promote the production of pro-inflammatory cytokines [33]. These results suggest that kefir may contribute to maintaining intestinal redox balance and increase antioxidant defense capacity, thereby reducing inflammatory responses.

Regarding modulation of the gut microbiota, the studies showed an increase in beneficial bacteria such as *E. coli*, *L. casei*, *B. fragilis*, *S. mitis*, *Lactococcus*, *Blautia*, *Akkermansia*, *Lachnospiraceae*, and a reduction in pathogenic microorganisms such as *S. typhi*, *K. aerogenes*, *E. cloacae*, *Bacteroides*, *Erysipelatoclostridium*, and *Turicibacter* [6, 10, 16, 18–21]. This shift in microbial profile may be related both to kefir’s composition and its ability to promote a gut environment more favorable to homeostasis.

Additionally, kefir showed a positive impact on intestinal barrier integrity. The increased expression of tight junction proteins such as ZO-1, occludin, and claudin-1 [17–21], along with the preservation of goblet cells [11, 13, 20] and improvement in colon length [6, 7, 12, 14, 16–19, 21], indicates a potential role for kefir in restoring intestinal architecture, which is essential for IBD management. Improvement in DAI [6, 11, 12, 17, 19, 21] also supports these findings.

The intestinal barrier protects the intestinal lumen. When damaged, it allows the entry of pathogens, antigens, and toxins, which activate the intestinal immune system, playing an important role in the pathogenesis of IBD. An altered intestinal barrier enables the circulation of activated immune cells, cytokines, and extracellular vesicles, which promote pro-inflammatory processes [34–36]. Furthermore, in IBD, there is a reduction in commensal microorganisms or a transition from commensal to pathogenic, which occurs under unfavorable conditions for the organism, generating dysbiosis. This, combined with the compromised intestinal barrier, exacerbates the inflammatory state that maintains the disease active and progressive [37]. Therefore, beneficial effects on the integrity of the intestinal barrier and microbiota diversity, such as those demonstrated by kefir, are essential in the context of IBD.

These experimental findings are also supported by clinical studies, as demonstrated by a randomized controlled trial involving IBD patients (*n* = 45) who consumed 400 mL of kefir for four weeks. In addition to an increase in fecal *Lactobacillus* and *L. kefiri* levels, participants showed improved hemoglobin and well-being scores, along with a significant reduction in inflammatory markers, such as CRP and erythrocyte sedimentation rate, and in bloating scores [38]. In another randomized controlled study involving soccer players, 200 mL of kefir for 28 days significantly increased beneficial microorganisms with anti-inflammatory effects, such as *Akkermansia muciniphila* and *Faecalibacterium prausnitzii* [39]. These findings reinforce the relevance of kefir as a modulator of the gut microbiota and a potential agent for reducing inflammation in clinical contexts.

We highlight these effects of kefir varied according to the administered daily dose (0,2 to 30 ml or 100 to 150 mg/kg), the type of kefir used (milk, water, or isolated microorganisms), the timing of the intervention (before or after colitis induction), and the duration (1 to 3 weeks) of the treatment. These factors directly influence the efficacy of the intervention, underscoring the need for future standardization in experimental protocols.

Most of the studies presented a moderate risk of bias, mainly due to the responses obtained in domains 3 to 7 of SYRCLE’s risk of bias tool. However, this appears to result from a common practice in experimental studies: failing to explicitly report items such as researcher blinding to experimental groups, random allocation of groups, and housing animals from different groups in separate cages. As a result, it is not possible to confirm whether these criteria (covered in domains 3 to 7) would receive a positive response, since the information is only implied. Therefore, reviewers were forced to classify them as ‘unclear’ due to the lack of explicit mention of blinding and allocation in the studies. Consequently, this led to a moderate risk of bias in most of the studies, even when the experimental design was of high quality. Thus, our review reinforces the importance of explicitly reporting methodological blinding and group allocation procedures in experimental studies, so that they can meet the criteria required by risk of bias assessment tools.

Therefore, the results reinforce the potential of kefir as an adjuvant agent in the treatment of IBD; however, they also highlight the need for well-designed clinical trials with larger sample sizes to confirm its efficacy in humans. Future studies should prioritize the standardization of kefir type and dosage, as well as consider different disease stages and treatment durations, in order to establish safe and effective clinical recommendations for its use. In this context, the promising findings, together with the robustness of the search process, underscore the importance of careful assessment of methodological quality and the risks of bias in the included studies.

## Conclusion

Kefir and its derivatives exert beneficial effects on inflammation, oxidative stress, gut health, and subsequently modulation of symptoms and pathology of IBD in animal models. Although the findings are promising, heterogeneity among study protocols and methodological limitations underscore the need for further studies, particularly in humans with IBD, to elucidate the mechanisms of kefir supplementation under controlled conditions in randomized clinical trials.

## Supplementary Information

Below is the link to the electronic supplementary material.

Supplementary file 1 (DOCX 11.5 KB)

Supplementary file 2 (DOCX 213 KB)

## Data Availability

No datasets were generated or analysed during the current study.
