# Supplementary material for: Kefir and Its By-Products Supplementation Reduces Inflammation and Oxidative Stress, Improves Intestinal Barrier Integrity, and Modulates the Gut Microbiota in Animal Models of Inflammatory Bowel Disease: A Systematic Review
Source: Probiotics Antimicrob Proteins. 2026 Mar 3;18(6):8182–208. doi: 10.1007/s12602-026-10948-5 (PMC13518411; doi:10.1007/s12602-026-10948-5)
Supplement: Supplementary file 1 [file 12602_2026_10948_MOESM1_ESM.docx]

**Kefir and its by-products supplementation reduces inflammation and oxidative stress, improves intestinal barrier integrity, and modulates the gut microbiota in animal models of inflammatory bowel disease - A systematic review.**

Maria Alice Spadarotto Neves 1, Rayanne Santos de Paulo 2, Josefina Bressan 2,3, Solange Silveira Pereira 1, Ana Claudia Pelissari Kravchychyn 1,3, Helen Hermana Miranda Hermsdorff 2,3*

1 Laboratory of Clinical Nutrition, Department of Nutrition and Health, Universidade Federal de Viçosa (UFV), Viçosa, MG, Brazil;

2 Laboratory of Clinical Analysis and Genomics, Department of Nutrition and Health, Universidade Federal de Viçosa (UFV), Viçosa, MG, Brazil;

3 Laboratory of Energy Metabolism and Body Composition, Department of Nutrition and Health, Universidade Federal de Viçosa (UFV), Viçosa, MG, Brazil.

*Correspondence author: Helen Hermana Miranda Hermsdorff, Department of Nutrition and Health, Universidade Federal de Viçosa (UFV), Viçosa, Minas Gerais - Brazil, Av. Peter Henry Rolfs, unnumbered - Campus Universitário, 36.570-900, Phone: +55 31 3612-5195, E-mail: helenhermana@ufv.br, ORCID: <https://orcid.org/0009-0007-1368-6020>.

### Supplementary table 2. Search strategies for studies included in analysis

| **PUBMED**  **May 8, 2025** | | **Search terms** | **Results** |
| --- | --- | --- | --- |
| **#1** | **Kefir** | kefir OR "kefir grains" OR "milk kefir" OR "water kefir" OR tibicos OR "sugary kefir" OR kefiran OR "kefir beverage" OR "probiotic kefir" OR "kefir culture" OR "lentilactobacillus kefiri" | 1.274 |
| **#2** | **Inflammatory Bowel Diseases** | "Inflammatory Bowel Diseases" [Mesh Terms] OR ‘’Inflammatory Bowel Disease’’ OR ‘’Bowel Diseases, Inflammatory’’ OR IBD OR "experimental inflammatory bowel disease" OR "irritable bowel disease" | 146.879 |
| **#3** | **Crohn disease** | "crohn disease" [Mesh Terms] OR ''Crohn's Disease'' OR ''Crohns Disease'' OR ''Crohn's Enteritis'' OR ''Inflammatory Bowel Disease 1'' OR ''Regional Enteritis'' OR ''Ileocolitis'' OR ''Terminal Ileitis'' OR ''Regional Ileitides'' OR ''Regional Ileitis'' OR ''Granulomatous Enteritis'' OR ''Enteritis, Regional'' OR ''Granulomatous Colitis'' | 76.965 |
| **#4** | **Ulcerative colitis** | ''Colitis, Ulcerative'' [Mesh Terms] ''Colitis Gravis'' OR ''Idiopathic Proctocolitis'' OR ''Inflammatory Bowel Disease, Ulcerative Colitis Type'' OR ''Ulcerative Colitis'' OR "colitis" OR "chronic ulcerative colitis" OR "ulcerosa colitis" OR "chronica colitis" OR "mucosal colitis" OR "ulcerative colorectitis" | 106.956 |
| **#5** | **Animal model** | "animal model" OR "experimental animals" OR "in vivo" OR animal OR animals OR rodentia OR rodent OR rodents OR murinae OR mouse OR mice OR mus OR musculus OR murine OR woodmouse OR rat OR rats | 9.141.443 |
| **#6** | **#1 AND #2** |  | 25 |
| **#7** | **#2 OR #3 OR #4** |  | 191.045 |
| **#8** | **#1 AND #7** |  | 32 |
| **#9** | **#1 AND #7 AND #5** |  | 23 |
| **Web of Science**  **May 8, 2025** | | | |
| **#1** | **Kefir** | ALL=(kefir OR "kefir grains" OR "milk kefir" OR "water kefir" OR tibicos OR "sugary kefir" OR kefiran OR "kefir beverage" OR "probiotic kefir" OR "kefir culture" OR "lentilactobacillus kefiri") | 2.810 |
| **#2** | **Inflammatory Bowel Diseases** | ALL=("Inflammatory Bowel Diseases" OR ‘’Inflammatory Bowel Disease’’ OR ‘’Bowel Diseases, Inflammatory’’ OR IBD OR "experimental inflammatory bowel disease" OR "irritable bowel disease") | 153.387 |
| **#3** | **Crohn disease** | ALL=("crohn disease" OR ''Crohn's Disease'' OR ''Crohns Disease'' OR ''Crohn's Enteritis'' OR ''Inflammatory Bowel Disease 1'' OR ''Regional Enteritis'' OR ''Ileocolitis'' OR ''Terminal Ileitis'' OR ''Regional Ileitides'' OR ''Regional Ileitis'' OR ''Granulomatous Enteritis'' OR ''Enteritis, Regional'' OR ''Granulomatous Colitis'') | 138.225 |
| **#4** | **Ulcerative colitis** | ALL=(''Colitis, Ulcerative'' OR ''Colitis Gravis'' OR ''Idiopathic Proctocolitis'' OR ''Inflammatory Bowel Disease, Ulcerative Colitis Type'' OR ''Ulcerative Colitis'' OR "colitis" OR "chronic ulcerative colitis" OR "ulcerosa colitis" OR "chronica colitis" OR "mucosal colitis" OR "ulcerative colorectitis") | 158.687 |
| **#5** | **Animal model** | ALL=("animal model" OR "experimental animals" OR "in vivo" OR animal OR animals OR rodentia OR rodent OR rodents OR murinae OR mouse OR mice OR mus OR musculus OR murine OR woodmouse OR rat OR rats) | 7.114.655 |
| **#6** | **#1 AND #2** |  | 34 |
| **#7** | **#2 OR #3 OR #4** |  | 272.946 |
| **#8** | **#1 AND #5 AND #7** |  | 30 |
| **EMBASE**  **May 9, 2025** | | | |
| **#1** | **Kefir** | ('kefir'/exp OR 'kefir' OR 'lentilactobacillus kefiri'/exp OR 'lentilactobacillus kefiri' OR kefir:ti,ab OR "kefir grains":ti,ab OR "milk kefir":ti,ab OR "water kefir":ti,ab OR tibicos:ti,ab OR "sugary kefir":ti,ab OR kefiran:ti,ab OR "kefir beverage":ti,ab OR "probiotic kefir":ti,ab OR "kefir culture":ti,ab) | 1.561 |
| **#2** | **Inflammatory bowel disease** | ('inflammatory bowel disease'/exp OR 'inflammatory bowel disease' OR 'IBD' OR 'experimental inflammatory bowel disease') | 272.152 |
| **#3** | **Crohn disease** | ('crohn disease'/exp OR 'crohn disease' OR 'colon crohn disease') | 129.279 |
| **#4** | **Ulcerative colitis** | ('ulcerative colitis'/exp OR 'ulcerative colitis' OR 'colitis'/exp OR 'colitis ' OR 'chronic ulcerative colitis' OR 'ulcerosa colitis' OR 'chronica colitis' OR 'mucosal colitis' OR 'ulcerative colorectitis') | 308.290 |
| **#5** | **Animal model** | ('animal model' OR 'experimental animals' OR 'in vivo' OR animal:ti,ab OR animals:ti,ab OR rodentia:ti,ab OR rodent:ti,ab OR rodents:ti,ab OR murinae:ti,ab OR mouse:ti,ab OR mice:ti,ab OR mus:ti,ab OR musculus:ti,ab OR murine:ti,ab OR woodmouse:ti,ab) | 5.094.313 |
| **#6** | **#1 AND #2** |  | 45 |
| **#7** | **#2 AND #3 AND #4** |  | 128.408 |
| **#8** | **#1 AND #2 AND #7** |  | 12 |
| **SCOPUS**  **May 10, 2025** | | | |
| **#1** | **Kefir** | TITLE-ABS-KEY (kefir OR "kefir grains" OR "milk kefir" OR "water kefir" OR tibicos OR "sugary kefir" OR kefiran OR "kefir beverage" OR "probiotic kefir" OR "kefir culture" OR "lentilactobacillus kefiri") | 2.968 |
| **#2** | **Inflammatory bowel disease** | TITLE-ABS-KEY ("inflammatory bowel disease" OR “IBD” OR "experimental inflammatory bowel disease" OR "irritable bowel disease") | 114.112 |
| **#3** | **Crohn disease** | TITLE-ABS-KEY ("crohn disease" OR "colon crohn disease") | 103.743 |
| **#4** | **Ulcerative colitis** | TITLE-ABS-KEY ("ulcerative colitis" OR "colitis" OR "chronic ulcerative colitis" OR "ulcerosa colitis" OR "chronica colitis" OR "mucosal colitis" OR "ulcerative colorectitis") | 157.664 |
| **#5** | **Animal model** | TITLE-ABS-KEY ( "animal model" OR "experimental animals" OR "in vivo" OR "animal" OR "animals" OR "rodentia" OR "rodent" OR "rodents" OR "murinae" OR "mouse" OR "mice" OR "mus" OR "musculus" OR "murine" OR "woodmouse") | 9.886.088 |
| **#6** | **#1 AND #2** |  | 62 |
| **#7** | **#2 OR #3 OR #4** |  | 258.711 |
| **#9** | **#1 AND #7 AND #5** |  | 26 |
